# Genetic and Environmental Associations Between Processing Speed and Executive Functions Across Adolescence and Established Adulthood

**DOI:** 10.1007/s10519-026-10271-3

**Published:** 2026-06-01

**Authors:** Mohini A. Karhadkar, Naomi P. Friedman, Chandra A. Reynolds, Robin P. Corley, Daniel E. Gustavson

**Affiliations:** 1 Institute for Behavioral Genetics, University of Colorado Boulder, Boulder, CO USA; 2 Department of Psychology and Neuroscience, University of Colorado Boulder, Boulder, CO USA

**Keywords:** Processing speed, Executive functions, Genetic influences, Cognitive trajectories, Twin study, Genetic correlation

## Abstract

Processing speed (PS), the rate at which an individual can perform basic cognitive tasks, and executive functions (EFs), cognitive abilities regulating thoughts and action, are key cognitive abilities related to important life outcomes. We investigated the relationship between PS and multiple EFs (a common factor and two factors specific to updating working memory and shifting mental sets) across adolescence (*M* age = 17.26 years, *SD* = 0.68) and established adulthood (*M* age 29.34 years, *SD* = 1.29). 797 individual twins (MZ = 428, DZ = 369) in the Colorado Longitudinal Twin Study completed 3 PS tasks and 6–9 EF tasks. PS_age16_ correlated strongly with PS_age29_ (*r* = .90) and Common EF (cEF) at age 17 (*r* = .70). Furthermore, cEF and Updating-Specific factors in late adolescence predicted new variance in PS_age29_ (*r*s = 0.13 to 0.16), but PS_age16_ did not predict new variance in cEF in established adulthood. PS and cEF were highly heritable at both ages (a^2^ > = 0.74) and genetically correlated at both time points (*r*_*a*_ = 0.70 to 0.73). PS was not significantly genetically correlated with the Updating- or Shifting-Specific factors in late adolescence, but was in established adulthood. Results indicate that PS and EFs are highly correlated yet distinct constructs. PS and EFs both demonstrate strong stability of genetic/environmental influences across nearly 15 years from late adolescence into established adulthood. It will be important for future developmental studies to understand why specific EF factors predict future variance in PS, but not the reverse.

**Supplementary Information:**

The online version contains supplementary material available at 10.1007/s10519-026-10271-3.

## Introduction

Processing speed (PS) and executive functions (EFs) are considered key domains of cognitive ability. PS reflects the rate at which an individual can perform basic cognitive tasks, and has been assessed in prior research using a variety of basic and more complex measures (Salthouse 2000). EFs are cognitive control processes that enable individuals to regulate thought and action in relation to goal-directed behavior (Miyake et al.^2000^; Diamond 2013; Gustavson et al.^2022^) and include measures of prepotent response inhibition, working memory updating, and mental set shifting. Both PS and EF have been linked to observed differences in cognitive development, age-related decline in other cognitive abilities, and psychiatric disorders, including ADHD, autism, schizophrenia, and substance use disorders (Friedman et al.^2008^; Diamond 2013; Gustavson et al. 2024). Although some studies question the separability of PS and EF in adolescents and adults (Salthouse 2005; Segura et al. 2025), prior research has typically demonstrated that the two broader cognitive abilities are highly correlated, yet still separable (Sanderson-Cimino et al. 2019; Friedman et al. 2008; Salthouse 2005). However, few studies have examined the phenotypic and genetic relationship between PS and EFs across time, including whether they contribute uniquely to one another’s development. PS and EFs are the first cognitive abilities to decline in normal aging, thus, understanding their relationship is crucial as it can inform theories of cognitive development and decline (Buckner 2004; Salthouse 2009; Friedman et al. 2016). Thus, investigating this relationship at different timepoints across the lifespan can help elucidate the extent to which PS and EFs share common and unique variance, and enables us to better understand the developmental interplay between these constructs. The current study therefore aimed to explore the associations between PS and EFs across late adolescence (ages 16–17) and established adulthood (age 29). First, we investigated the stability of PS ability between late adolescence and established adulthood. Second, we examined whether PS and EFs in adolescence predicted new variance in the other construct in adulthood and decomposed the genetic and environmental etiology of the cross-sectional associations between PS and EFs.

### Processing Speed: Definitions and Earlier Work

Carroll (1993) defined ‘Processing Speed’ as a Stratum II ability, nested within their three stratum model of the psychometric structure of intelligence. It is now broadly referred to as Cattell-Horn-Carroll theory (CHC; Jewsbury et al. 2016; Schneider and McGrew 2018). The CHC model suggests that PS is more specific than the concept of general intelligence, but still encompasses a wider set of abilities than an individual task measure of cognition (i.e., Perceptual Speed, Reading/Writing Fluency). Researchers have defined PS, or more generally ‘speed’, as a range of processes, including but not limited to, *decision speed* (moderately complex content), *perceptual speed* (elementary content), *psychomotor speed* (i.e., repetitive finger tapping), and *psychophysical speed* (decision accuracy based upon audiovisual stimuli) (Salthouse 2005). Despite differences in combinations of speed assessments used in research, there is evidence for robust common variance across different measures of speed in adolescence (Friedman et al.^2008^), and midlife (Sanderson-Cimino et al. 2019). Moreover, Sanderson-Cimino et al. (2019) suggested that combining measures of sub-categories of PS (e.g., perceptual speed, psychomotor speed, etc.) into a latent variable model may more accurately capture an individual’s cognitive ability, especially at the level genetic influences. As we describe in more detail later, earlier studies have demonstrated that genetic influences account for a substantial proportion of the variability in PS in both adolescence and midlife (Friedman et al.^2008^; Sanderson-Cimino et al. 2019). However, studies have not yet estimated the heritability of PS in established adulthood using a twin study design, or examined the stability of genetic and environmental influences on PS between late adolescence and established adulthood.

### The Unity and Diversity of Executive Functions

EFs have been researched extensively in the fields of cognitive psychology and behavioral genetics (Diamond 2013; Friedman and Miyake 2017a, b). Research on EFs has broadly examined three domains: inhibitory control, working memory, and cognitive flexibility (Diamond 2013). The theoretical framework employed here focuses on the most commonly studied facets of each domain that have been the focus on the behavioral genetic and latent work on EFs, on which the current builds (Miyake et al.^2000^; Friedman et al.^2008^): prepotent response inhibition (Inhibition; representative of inhibitory control, specifically the ability to inhibit dominant, automatic, or prepotent responses); updating working memory representations (Updating; reflecting the ability to monitor, manipulate, and replace information in working memory); and mental set shifting (Shifting; a specific aspect of cognitive flexibility involving the ability to flexibly switch back and forth between tasks or mental sets) (Friedman et al.^2008^; Friedman and Miyake 2017a, b; Gustavson et al.^2022^). A seminal study of these three constructs demonstrated that latent variables capturing *Inhibition*, *Updating*, and *Shifting*, shared a moderate proportion of variance, thus showing “unity” between the EFs. However, these correlations were significantly less than 1.0, indicating that the three EFs are nonetheless distinct processes, which refers to the “diversity” of EFs (Miyake et al.^2000^).

Recent frameworks of EF, including that utilized here, highlight that variance in these classic EF domains can be reconceptualized (e.g., using a bifactor model) into three other constructs (Friedman and Miyake^2012^, 2017a, b). Specifically, a ‘Common EF’ latent variable can be used to capture variance across all types of EF tasks, and is thought to reflect general goal-management processes that underlies performance in a multitude of situations (Engelhardt et al. 2016; Friedman and Miyake 2017a, b). Additional latent variables can be used to capture variance in working memory updating (Updating-specific) and task-set shifting (Shifting-specific) tasks not already captured by Common EF, and are thought to reflect working memory and memory retrieval and the ability to flexibly replace goals, respectively (Friedman and Miyake 2017a, b). This conceptualization of EF performance is particularly useful given that EFs appear to be primarily associated with other cognitive, mental health, and substance use outcomes through Common EF variance rather than through Updating-specific or Shifting-specific variance (Gustavson et al. 2015,2024), though Updating-specific has been linked to intelligence tests above and beyond the association between Common EF and intelligence (e.g., Friedman et al.^2008^; Gustavson et al.^2022^). Finally, work using this model has not identified evidence for Inhibition-specific variance, suggesting that individual differences in prepotent response inhibition are closely related to the variance captured by Common EF (Friedman and Miyake 2017a, b; Freis et al. 2022; Gustavson et al.^2018^).

### Overlap Between Processing Speed and Executive Functions

PS and EFs are among the first cognitive abilities to exhibit normal age-related decline, with abilities typically reported to peak in early to mid-adulthood (Buckner 2004; Salthouse 2009; Hartshorne and Germine 2015; Friedman et al.^2016^). This range of peaks implies that investigating changes in cognitive abilities between late adolescence and established adulthood is key to understanding early age-related cognitive decline. Thus, examining these associations in established adulthood, shortly after the peak of these abilities, offers a valuable point of comparison with prior research focused on children and adolescents, during which EFs, in particular, are still developing.

PS and EFs are reported to be highly correlated with one another. Cepeda et al. (2013), for example, suggest that PS tasks could necessitate executive processes (i.e., goal maintenance, working memory, decision-making), thus confounding any distinction between PS and EFs. Younger individuals may particularly require more executive control to carry out simpler PS tasks than established adults who have had more experience tapping into the necessary processes. Cepeda et al. (2013) also highlight that specific abilities that contribute to completing PS tasks are likely to have different developmental trajectories, and thus, patterns of development and decline between PS and EFs depend on the PS measures selected for research. Indeed, McAuley and White (2011) showed that PS, response inhibition, and working memory were statistically distinguishable at the age of 6, and that this distinction persisted until at least the age of 24. They found that PS accounted for significant variance in both working memory and a single indicator of inhibition, but emphasized that the constructs are not interchangeable. These findings support the notion that PS facilitates EF development, potentially by enabling faster goal maintenance or contextual processing, though this requires further testing with more robust models of EFs.

Other work has suggested that PS and EF inherently tap into one another, and may even be indistinguishable (Salthouse 2005). Following this, near-identical correlations have been observed in studies that did not adequately control for baseline speed conditions in EF tasks. This is a known issue in EF literature (i.e., the ‘task impurity problem’), as it is difficult to study cognitive control in isolation, as they often occur in the context of lower-level cognitive processes such as PS. Thus, reaction time-based EF tasks typically involve adjusting trials with high EF demands (e.g., incongruent trials on the Stroop task where the text color does not match the word name) based on trials with low or no EF demands (e.g., naming colors of strings of Xs or asterisks). Nevertheless, even when adjusting for baseline conditions, moderate-to-strong correlations between PS and EFs persist. For example, Friedman et al. (2008) found that ‘Perceptual Speed’ was significantly genetically correlated (*rG*s *=* 0.19 to 0.63) with two EF factors (Common EF, and Updating-Specific) during late adolescence (ages 16–17). Such findings are indicative of a meaningful overlap in the underlying genetic architecture of PS and EFs, while also showing that the two constructs are separable. However, it will be important to examine whether these associations generalize to other life stages, such as established adulthood.

Finally, while much has been documented about the correlations between PS and EFs, the directionality of their relationship remains unclear. An important question yet to be explored is whether PS and EFs predict unique variance in each other across key developmental stages of the lifespan. This is particularly relevant given that both PS and EFs are among the first cognitive abilities to show age-related differences or decline (Friedman et al.^2016^; Salthouse 2009). Prior research by Schaie (1989) found only marginal evidence that speed predicts future cognitive ability, but not EFs, in adulthood when using cross-lagged correlational analysis.

### Leveraging Behavior Genetics Approaches to Understand the Overlap Among PS and EF

Twin studies are often utilized in the field of behavioural genetics to quantify how genetic and/or environmental influences explain the proportion of variance in a trait, or the covariance among traits. Specifically, data from monozygotic (MZ, identical) and dizygotic (DZ, fraternal) twins have been used to estimate the extent to which individual differences in PS and EFs are influenced by additive genetic (A), shared environmental (C) and non-shared environmental (E) influences (Friedman et al.^2008^). Additive genetic (A) influences comprise the cumulative effects of individual genes affecting the complex trait in question. Shared environmental (C) influences typically reflected spaces shared by twins; for example, familial environment (when twins are reared together), sociodemographic factors (family income, urbanicity, education), as well as peri-natal conditions. Moreover, the same environment can contribute to both the shared and non-shared environmental (E) influence estimates. For example, whilst both twins in a pair may attend the same school, their individual experiences of education may vary based upon non-genetic factors. Thus, aspects of the schooling environment experienced or perceived similarly across twins will be captured by the C estimate while individual experience are captured by the E estimate.

Decomposing phenotypic variance into its genetic and environmental components provides a more comprehensive understanding of the factors contributing to individual differences, offering greater clarity on associations than phenotypic correlations alone. Understanding these contributions is essential for advancing knowledge on the co-development of PS and EFs. Prior research has demonstrated that variance in both PS and EFs are primarily explained by genetic influences, with some non-shared environmental contributions, but not shared environmental influences (Friedman et al.^2008^). Moreover, genetic influences account for much of the stability in EFs (Friedman et al.^2016^), but we know less about the stability of genetic and/or environmental influences on PS across key developmental stages in the lifespan, such as from late adolescence and established adulthood. Bringing previous research together, we expected that genetic influences would contribute substantially to the stability of PS between adolescence and adulthood, and that PS and EFs would remain at least moderately genetically correlated with one another in established adulthood. Moreover, if observed genetic (and environmental) correlations are also less than 1.0, this would provide stronger evidence that PSs and EFs represent distinct constructs. Furthermore, we may expect to observe changes in environmental contributions to these constructs, with shared environmental influences being potentially more pertinent in adolescence (i.e., proximity to family, education systems) than in established adulthood, where non-shared environmental variance on components may increase.

### Current Study

The present study examined the relationship between PS and the three EFs using data from the Colorado Longitudinal Twin Study (LTS), which included PS and EF assessments in adolescence (mean age 16–17, LTS) and established adulthood (mean age 29). First, we explored the stability of PS between adolescence and adulthood as well as the phenotypic overlap between PS and EFs at both assessments. Second, we evaluated whether PS and EFs predicted one another longitudinally, controlling for baseline measurements. Third, we utilized twin analyses to inform the stability of genetic and environmental influences on PS between late adolescence and established adulthood and to estimate the magnitude of genetic and (shared/non-shared) environmental influences on PS and EFs at both time points. In these analyses, we also decomposed cross-sectional associations between PS and EF factors into their genetic, shared environmental, and non-shared environmental components. We predicted that PS would be associated primarily with Common EF (rather than Updating-specific or Shifting-specific factors), and that this association, at both time points, would be driven by genetic influences.

## Methods

### Participants

Participants were 797 individual twins (female = 417) from the Colorado Longitudinal Twin Study (LTS) with data for PS (*n* = 795) and EFs (*n* = 779) during late adolescence (i.e., ages 16–17), and/or data for PS (*n* = 652) and EFs (*n* = 629) during established adulthood (i.e., at ~ age 29); 524 of these participants have complete data at all time points. These data include 395 complete twin pairs (and 7 individual twins); 213 of these complete pairs are monozygotic (MZ, identical) twins, and 182 pairs are dizygotic (DZ, fraternal) twins. All participants were included even if they only participated in one assessment, as they are still informative for heritability estimates and cross-sectional associations.

LTS participants, born between 1984 and 1990, were recruited in Colorado, and were followed from birth (Corley et al. 2019). Participants identified as White (91.6%), Hawaiian, Pacific Islander, American Indian, or Alaskan (1.6%), more than one race (5.4%), or unknown or unreported race (1.4%) (Wadsworth et al. 2019); Hispanic individuals comprised 9.7% of the sample (Corley et al. 2019). Zygosity was determined through repeated tester ratings and genotyping.

In late adolescence, participants completed PS tasks at around age 16 (*M* = 16.27, SD = 0.68) and EF tasks at around age 17 (*M* = 17.26, SD = 0.64). In adulthood, participants completed PS tasks as part of the Colorado Adoption/Twin Study of Lifespan Behavioral Development and Cognitive Aging (CATSLife) project (Wadsworth et al. 2019) (*M*_*age*_ = 29.34, SD = 1.29). EF tasks for some individuals (*n* = 652) were completed as a part of the same set of CATSLife assessments, however a subset of participants (*n* = 485) completed 3 of the EF tasks (antisaccade, keep track, number-letter switching) in an MRI scanner and the remaining 3 EF tasks outside the scanner as part of a separate assessment (LTS EF-MRI) *M* = 0.17 years (SD = 0.77) before the primary CATSLife assessment (Corley et al. 2019; Gustavson et al.^2022^). Prior work describes the harmonization procedure between the CATSLife and LTS EF-MRI assessments (Gustavson et al.^2022^), which leveraged some individuals who completed EF tasks at both assessments to assist in harmonization (*n* = 175). When participants had both assessments, we prioritized the CATSLife assessment, except for the letter memory task, which was the only task to show evidence for practice effects, so we used letter memory data from the LTS EF-MRI assessment (Gustavson et al.^2022^).

### Measures

#### Processing Speed

PS was assessed with three tasks at both time points. The Colorado Perceptual Speed (CPS) task is described as part of Kent and Plomin’s (1987) Colorado Battery of Specific Cognitive Abilities with tasks details in DeFries et al. (1981). The Subtraction and Multiplication task was from the Education Testing Service Kit of Factor-Referenced Cognitive Tests (French et al. 1976). Finally, we used the Digit Symbol subtest of the Wechsler Adult Intelligence Scale III (Wechsler 1997). For PS measures at both timepoints, we constructed the CPS and SAM variables based on the proportion of correct answers given in the respective task set.

The CPS had two sections, each of which contained 30 items (DeFries et al., 1981). Each item consisted of a target set of four letters or numbers on the left, and four sets on the right, one of which was the same as the stimulus set on the left. The participant was required to indicate which of the sets on the right is the same as the target; the dependent measure was the average number (i.e., out of 30) of sets correctly selected in 1 min. The ‘Subtraction and Multiplication’ task assessed the participant’s ability to perform basic arithmetic operations, specifically simple two-digit subtraction problems and two-digit by one-digit multiplication problems. The dependent measure was the number of correct answers completed within 2 min. In the Digit Symbol subtest, the participant was first presented with a key containing digits paired with abstract symbols. They were then shown a set of numbers/symbols that they had to match the corresponding number/symbol to, as indicated by the initial key. The dependent measure was the number of correct responses, within 2 min.

#### Executive Functions

Assessment of EF at the age 17 (Friedman et al.^2008^) and age 29 assessments (Gustavson et al.^2022^) have been described in detail in prior studies but are summarized here. At age 17, LTS participants completed nine computerized tasks designed to assess response inhibition, working memory updating, or mental set shifting. At age 29, a subset of six tasks (*Antisaccade*,* Stroop*,* Keep Track*,* Letter Memory*,* Number Letter*, and Category Switch) was included which had the highest factor loadings on their respective factors in earlier studies (Gustavson et al.^2022^).

##### Inhibition

Prepotent response inhibition is the ability to deliberately control or inhibit automatic, or dominant responses (Miyake et al.^2000^). At the age 17 assessment, the three tasks used to measure response inhibition were the antisaccade, Stroop, and stop signal tasks. In the antisaccade task, adapted from Roberts et al. (1994), participants saw a cue (a black square) flash on one side of the screen, and had to avoid the reflexive tendency to saccade to these cues in time to see a target stimulus (a digit from 1 to 9) before the digit was masked. The dependent measure was the accuracy of target identification on the antisaccade blocks. In the Stroop task, participants named the color of strings of asterisks (neutral blocked trials) or color words (printed in different colors, e.g. *RED* in green; incongruent blocked trials) (Stroop 1935). The incongruent trials required individuals to avoid their prepotent tendency to read the words. The dependent measure was the mean reaction time (RT) on incongruent color word trials minus the mean RT on asterisk trials. In the stop signal task, participants saw green arrows on the screen and responded (left or right) as per the direction of the arrow (Logan et al. 1997). If the arrow turned red shortly after it was displayed (occurred 25% of the time), the participants were instructed to stop their response. The dependent measure was the stop-signal RT, which is the estimated time at which the stopping process finishes. At the age 29 assessment, only the antisaccade and Stroop tasks were measured.

##### Updating

Working memory updating is the ability to monitor incoming information and, when relevant, add newly relevant items into, and remove no-longer-relevant items from, working memory (Miyake et al.^2000^). The updating tasks (*Keep Track*, *Letter Memory*, and *Spatial 2-back*) required participants to monitor and manipulate the contents of working memory. The *Keep Track* task, adapted from Yntema (1963), had participants looking at a list of 15–25 words drawn from 6 categories (e.g. animals, metals, countries). They had to remember the most recently presented words from each of 2–5 target categories. The dependent measure was the proportion of total words recalled across 16 trials (the accuracy of recalling target words). In *Letter Memory*, participants saw a series of letters that were unpredictable in length (9, 11, or 13 letters long), with each letter being displayed for 3 s. The participants were required to continuously rehearse aloud the last 4 letters they had seen (including the current letter shown to them). The dependent measure was the proportion of sets correctly rehearsed across all the letters presented in 12 trials. In the *Spatial 2-back task*, participants saw 12 squares that flashed on the screen one at a time (Friedman et al.^2008^). After each flash, participants responded with a yes or no, as to whether that specific spatial position on the screen was the same that flashed 2 trials ago. The dependent measure was the mean *z*-score of arcsine accuracy (yes or no responses). In the LTS-EF assessments, *Keep Track*,* Letter Memory*, and *Spatial 2-back* were included, whereas in the CATSLife assessments, only *Keep Track* and *Letter Memory* were measured.

##### Shifting

Set shifting is the ability to flexibly switch back and forth between multiple task sets or operations (Miyake et al.^2000^). The tasks (*Number Letter*,* Category Switch*, and *Color Shape*) required participants to switch between two subtasks that used the same two button-box responses, according to a cue that appeared just before the stimulus and remained on the screen with the stimulus until they responded. Half of the trials required repeating the task from the prior trial (e.g. judge color after having just judged color), and half required switching tasks (e.g. judge color after having just judged shape). The dependent measure for all tasks was the ‘local switch cost’: the average RT for switch trials minus the average RT for repeat trials. In the LTS-EF assessments, *Number Letter*,* Category Switch*, and *Color Shape* were included, whereas in the CATSLife assessments, only *Number Letter* and *Category Switch* were measured.

In the *Number Letter* task, adapted from Rogers and Monsell (1995), participants saw a number-letter pair (e.g. *E7*) presented in one quadrant of a box on the screen, and were required to categorize the pair as having an even or odd number (if the pair appeared in the top 2 quadrants), or as having a vowel or a consonant (if the pair appeared in the bottom 2 quadrants). In the *Category Switch* task, adapted from Mayr and Kliegl (2000), participants categorized a word (e.g. *lizard*) as describing something that is smaller or bigger than a soccer ball, or living or non-living, depending on a cue symbol (crossed arrows or a heart) that appeared above it. In the *Colour Shape* task, adapted from Miyake et al. (2004), participants were presented with a colored shape, and were asked to categorize the color of the shape as red or green, or the shape as a circle or a triangle, depending on a cue letter (*C*, for color, or *S*, for shape) that appeared above it.

### Data Analysis

#### Phenotypic Analyses

Phenotypic analyses were conducted using the ‘lavaan’ package in R version 4.4.1, and model fit of the latent variables was determined using the RMSEA (root mean squared error of approximation) and the CFI (comparative fit index). Good fitting models had RMSEA values < 0.06 and CFI values > 0.95 (Hu and Bentler 1999). These analyses accounted for the fact that observations within the same family are not independent of one another using the command [cluster = “family”] when running structural equation models. The latent variable modelling also utilized Full Information Maximum Likelihood estimation, to estimate model parameters where missing, rather than excluding cases with missing values. For all associations, we report *p*-values of significance and standard error-based 95% confidences intervals (CIs).

For all dependent measures based on reaction times, scores excluded trials with errors. In the shifting tasks, trials after errors were also excluded, as switch versus repeat trial types are ambiguous if following errors (Gustavson et al.^2022^). All dependent measures based on RT were reversed scored, so that lower values were indicative of worse performance (i.e., Stroop, Number Letter, Colour Shape, Category Switch, and Stop Signal). All dependent measures (including RT and accuracy-based measures) were residualized for sex and age prior to analysis – this adjustment was based on some of the EF tasks (e.g., Antisaccade, Category Switch) demonstrating sex effects in previous research (Friedman et al.^2011^). All EF variables were z-scored prior to analyses. PS variables in adolescence were *z*-scored prior to analyses and PS variables in established adulthood were scaled with respect to the means and SDs from the PS variables in late adolescence, to allow for invariance testing.

Preliminary analyses for measurement invariance were conducted for the PS latent variable across both time points (age 16, age 29) to ensure that any inferences we made about changes between the latent variables reflected change in PS, rather than changes in measurement and/or participant characteristics. Invariance testing was not conducted for the EF models, as different sets of assessments were used in established adulthood, as compared to late adolescence (and some parameters changed in the retained tasks). For the PS factors, we tested increasingly restrictive models, which included testing for invariance in factor loadings, intercepts and residual variances. Model fit statistics (including χ^2^, df, RMSEA, CFI) and model comparisons (Δχ^2^) are present in Supplemental Table S1. The model with the most constraints implemented (in which factor loadings, intercepts, and residual variables were equated) did not fit significantly worse than the model with configural invariance (Δχ^2^ (7) = 12.898, *p* =.075). This indicates that PS was measured consistently across the two time points, and thus, these strict constraints were included in all phenotypic and longitudinal genetic models of PS.

#### Genetic Analyses

Genetic analyses were also conducted in R, using the ‘OpenMx’ package (Neale et al. 2016), and were based on standard assumptions of the classical twin study design (Neale and Cardon 1992). OpenMx also utilizes Full Information Maximum Likelihood estimation, and estimates likelihood-based 95% confidence intervals. We examined additive genetic influences (A), shared environmental influences (C), and nonshared environmental influences (E), that contribute to the total variance of each of the observed measures and latent variables. Additive genetic influences correlate 1.0 in MZ twins (MZTs), because they share 100% of their alleles; and they correlate 0.5 in DZ twins, who share, on average, 50% of their alleles. Shared environmental influences (C) are indicative of factors such as the common familial environment shared by twins, siblings, and parents alike; hence, they correlate 1.0 for both sets of twins. Non-shared environmental influences (E) account for the remaining environmental factors that are not shared between family members (including twins), and by definition, make the twins differ from one another; thus, they do not correlate in either MZ or DZ twins.

We calculated the variances for each of these components (i.e., a^2^, c^2^, and e^2^) in the latent variable models, as well as the correlations between variances for PS and EFs within and across time points. We did not estimate the shared environmental correlations (*r*_C_) between PS and EFs at either time point, as c^2^ was estimated at zero for all three EF factors (‘Common EF’, ‘Updating-Specific’, and ‘Shifting-Specific’) in earlier work (Friedman et al.^2008^; Gustavson et al.^2022^). For the same reason, we did not estimate *r*_E_ (the non-shared environmental correlation) between PS and the ‘Updating-Specific’ factor at either time point. We also decomposed residual variances in each measures into a^2^, c^2^, and e^2^ influences, and allowed correlations among all residual PS factors in the longitudinal PS model (though residual c^2^ correlations were only estimated for the Subtraction and Multiplication task because this was the only measure of PS to show evidence for shared environmental influences).

#### Sample Overlap

Friedman et al. (2008) reported on the association between a ‘Perceptual Speed’ latent variable and EFs, as well as the association between ‘WAIS IQ’ and EFs, in the LTS sample at age 17. This perceptual speed factor was based on one of the same tasks we used (CPS), but also based on the *Identical Pictures* and *Hidden Pattern*s tasks from the Educational Testing Service battery, which were unavailable at the age 29 assessment. We conducted a preliminary phenotypic investigation of five measures loaded onto a ‘PS’ latent variable at age 16 (*hidden patterns*,* identical pictures*,* subtraction and multiplication*,* CPS*, and *WAIS digit symbol*). This SEM diagram is available in the supplement (Fig. S1). ‘CPS’, ‘Subtraction and Multiplication’, and ‘WAIS Digit Symbol’ were all assessed at both points, and their factor loadings on the PS latent variable (.62 to.79) were similar to those of ‘Identical Pictures’ and ‘Hidden Patterns’. Hence, we constructed our PS latent variable using only these three measures, and analyzed PS at two time points (mean age 16, and mean age 29). We also previously reported the heritability of EF factors at age 29 (Gustavson et al.^2022^), but this earlier study did not include any measures of PS.

## Results

### Descriptive Statistics

Descriptive statistics for all PS and EF assessments are below (Table 1). Phenotypic correlations among all raw measures (Table S2) are displayed in the supplement.

**Table 1:** Descriptive statistics for all study measures

Measures	*N*	M	SD	Range	Skewness	Kurtosis	rMZ	rDZ
Longitudinal Twin Study, LTS								
Age (at PS assessments)	797	16.27	0.68	4 (16, 20)	2.75	7.27		
Colorado Perceptual Speed	795	18.93	3.92	24.5 (5, 29.5)	−0.03	0.12	0.715	0.304
Subtract and Multiply	795	15.87	6.97	43.5 (1, 44.5)	0.67	0.67	0.686	0.501
Digit Symbol, WAIS III	795	10.35	2.68	15 (3, 18)	0.05	−0.25	0.626	0.295
Age (at EF assessments)	797	17.26	0.64	3.57 (16.51, 20.08)	1.69	3.06		
Antisaccade	779	1.04	0.20	1.1 (0.47, 1.57)	−0.12	−0.27	0.526	0.145
Stroop	759	274.4	90.11	488.43 (−0.22, 488.21)	−0.58	0.17	0.473	0.275
Stop Signal	741	357.7	62.56	338.33 (150.62, 488.95)	−1.12	1.48	0.478	0.072
Keep Track	774	0.94	0.18	1.11 (0.38, 1.49)	0.30	0.54	0.512	0.251
Letter Memory	785	1.09	0.25	1.19 (0.38, 1.57)	0.29	−0.21	0.570	0.188
Spatial 2-back	777	1.17	0.17	0.92 (0.65, 1.57)	−0.92	1.62	0.285	0.143
Number Letter (No Switch RT)	776	578.2	183.2	937.03 (−14.01, 923.02)	−1.03	1.09	0.497	0.287
Category Switch (No Switch RT)	766	531.4	181.4	933.37 (−34.36, 899.01)	−0.97	0.90	0.515	0.314
Color Shape (No Switch RT)	768	388.5	189.4	1111.82 (−196.05, 915.77)	−0.75	0.72	0.338	0.188
CATSLife								
Age (at PS assessments)	652	29.34	1.29	6.53 (28.05, 34.58)	1.26	1.20		
Colorado Perceptual Speed	652	20.12	2.03	25 (5, 30)	0.08	−0.06	0.692	0.346
Subtract and Multiply	648	14.28	7.32	53 (1, 54)	1.18	2.37	0.748	0.452
Digit Symbol, WAIS III	648	11.65	2.83	15 (4, 19)	−0.03	−0.24	0.661	0.386
Age (at EF assessments)	652	29.33	1.29	6.53 (28.05, 34.58)	1.28	1.26		
Antisaccade	629	73.12	13.46	78.91 (20.83, 99.75)	−0.47	−0.02	0.569	0.142
Stroop	649	−149.2	77.20	516.67 (−426.38, 90.29)	−0.78	1.14	0.381	0.258
Keep Track	649	70.85	8.71	70.59 (26.63, 97.22)	−0.93	2.60	0.475	0.150
Letter Memory	649	73.20	14.13	74.27 (25.73, 100)	−0.11	−0.072	0.674	0.313
Number Letter (No Switch RT)	638	−248.2	152.38	1010.59 (−928.96, 81.9)	−1.28	2.68	0.577	0.287
Category Switch (No Switch RT)	645	−184.2	153.40	903.16 (−800.11, 103.05)	−1.14	1.40	0.624	0.133

All raw PS and EF measures, displayed here, were scaled and residualised on Sex and Age prior to analysis. Stroop, Number Letter, Color Shape, Category Switch, and Stop Signal were reverse scored prior to analyses.

*PS* Colorado Perceptual Speed, *WAIS* Wechsler Adult Intelligence Scale, *EF* Executive function, *RT* Reaction time, *CATSLife* Colorado Adoption/Twin Study of Lifespan Behavioral Development and Cognitive Aging.

### Phenotypic Analyses

**Fig. 1:**
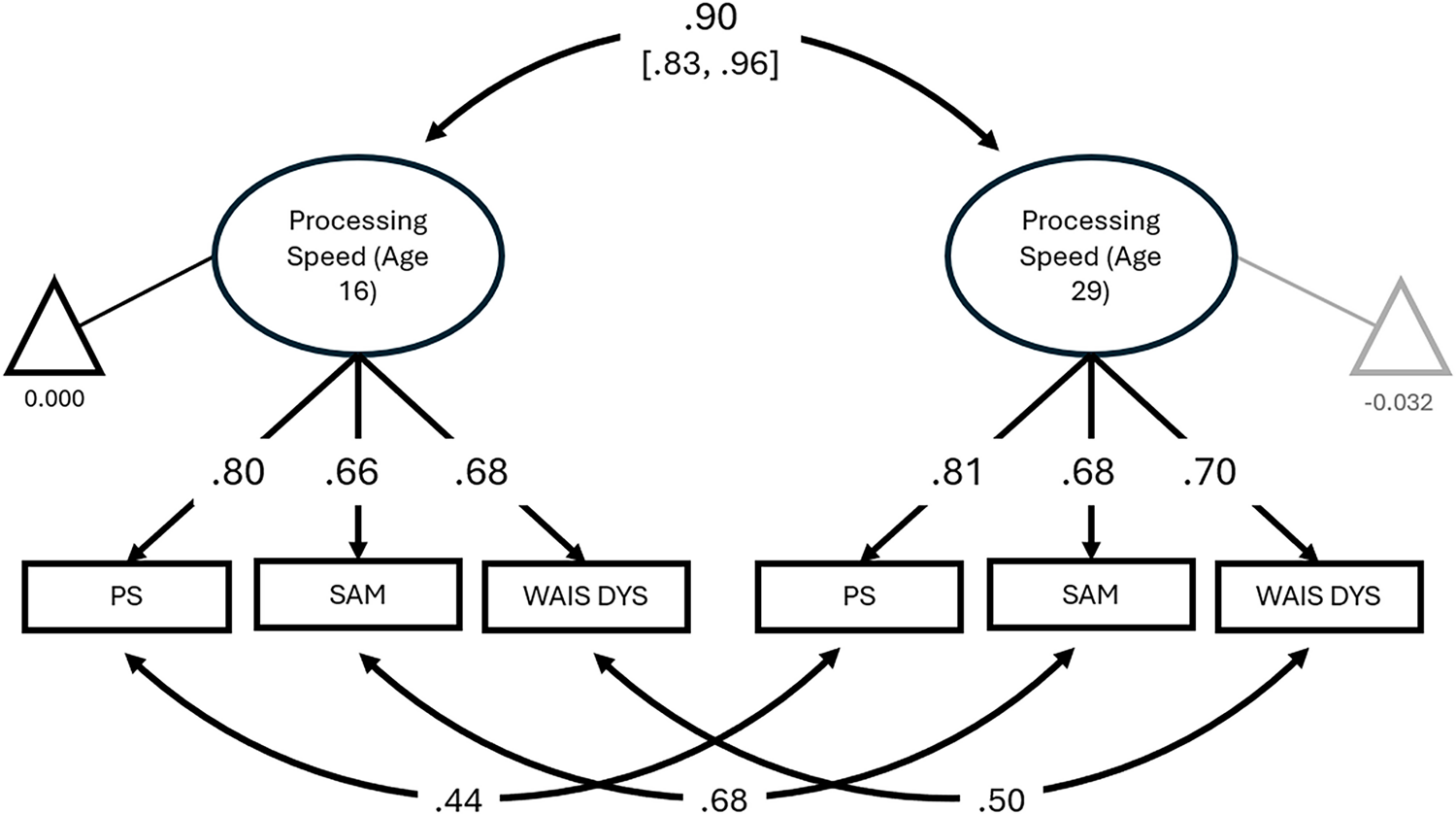
Longitudinal phenotypic relationship between latent variables of processing speed. Variance explained by latent variables (ovals) are computed by squaring factor loadings on measured variables (rectangles). Factor loadings, intercepts, and residual variances for all observed measures were equated across time in a strong invariance model. The latent variable mean (triangle) for PS_age16_ was fixed to 0, whilst the latent mean for PS_age29_ was freely estimated. Significant paths are displayed in black text, with solid black arrows (*p* <.05), non-significant paths are displayed in grey text, with grey lines (*p* >.05). Model fit: χ^2^ (12) = 28.027, *p* =.005. CFI = 0.993, RMSEA = 0.041). Standardized results are shown. PS = Colorado Perceptual Speed, SAM = Subtract and Multiply, WAIS DYS = Wechsler Adult Intelligence Scale Digit Symbol

Figure 1 displays the latent factor model of Processing across late adolescence and established adulthood, using data from three task indicators: the Colorado Perceptual Speed Test (PS), Subtract and Multiply from the Cognitive Abilities Battery (SAM), and the Digit Symbol subtest from the Wechsler Adult Intelligence Scale (WAIS DYS). Each assessment loaded significantly onto the latent PS factor at their respective time point (0.66 − 0.82), supporting the construct validity of the model. The correlation between the two latent PS factors reflects the high stability of PS from adolescence to established adulthood (*r* =.90, 95% CI [0.83, 0.96]). We constrained the factor loadings, intercepts, and residual variances of the observed measures in this stringent invariant phenotypic model. The latent mean of Processing Speed at age 29 was not significantly different from the latent mean of PS at age 16 (*M* = −0.032, *p* <.05), indicating that PS performance in established adulthood has not yet significantly decreased from adolescence.

#### Relationship Between PS and EFs

We next examined correlations between PS, Common EF, Updating-Specific, and Shifting-Specific abilities in late adolescence and established adulthood to characterize their phenotypic relationships across crucial periods of cognitive development. This latent variable correlation matrix is displayed in Table 2, with a full SEM path diagram provided in the supplement (Fig. S2).

**Table 2:** Latent variable correlation matrix

	PS16	cEF17	Up17	Sh17	PS29
PS16	1				
cEF17	**0.68 [0.62**,** 0.75]**	1			
Up17	**0.24 [0.16**,** 0.31]**	-	1		
Sh17	−0.04 [−0.09, 0.11]	-	-	1	
PS29	**0.90 [0.77**,** 1]**	**0.68 [0.61**,** 0.75]**	**0.32 [0.25**,** 0.39]**	−0.04 [−0.09, 0.13]	1
cEF29	**0.61 [0.54**,** 0.67]**	**0.89 [0.81**,** 0.97]**	-	-	**0.67 [0.59**,** 0.75]**
Up29	**0.23 [0.16**,** 0.30]**	-	**0.96 [0.89**,** 1]**	-	**0.42 [0.35**,** 0.50]**
Sh29	0.00 [−0.06, 0.06]	-	-	**0.71 [0.63**,** 0.79]**	0.02 [−0.03, 0.08]

Statistically significant estimates are displayed in bold text (*p* <.05). ‘-’ indicates correlations fixed to 0. Model fit: χ^2^ (163) = 386.978, *p* <.001. CFI = 0.957, RMSEA = 0.042 [90% CI (0.036, 0.047)].

*PS* Processing speed, *cEF* Common EF, *Up* Updating-Specific, *Sh* Shifting-Specific.

Similar to PS, all three EF factors were highly correlated with themselves between ages 17 and 29 (*r* =.71 to 0.96). In late adolescence, PS was strongly positively correlated with Common EF (*r* =.68, 95% CI [0.62, 0.75]), modestly positively correlated with Updating-Specific abilities (*r* =.24, 95% CI [0.16, 0.31]), and non-significantly correlated with Shifting-Specific abilities (*r* = −.04, 95% CI [−0.09, 0.11], *p* >.05). In established adulthood, we again observed a high phenotypic correlation between PS and Common EF (*r* =.67, 95% CI [0.59, 0.75]), a moderate correlation between PS and Updating-Specific abilities (*r* =.42, 95% CI [0.35, 0.50]), and a nonsignificant association between PS and Shifting-Specific abilities (*r* =.02, 95% CI [−0.03, 0.08], *p* <.05). These findings indicate that PS and Common EF are consistently associated with one another across late adolescence and adulthood, as are PS and Updating, whilst associations between PS and the Shifting-Specific factor remained non-significant at both time points. In all cases, 95% CIs did not overlap with 1.0, indicating PS was significantly separable from Common EF and Updating-Specific factors. 

**Fig. 2:**
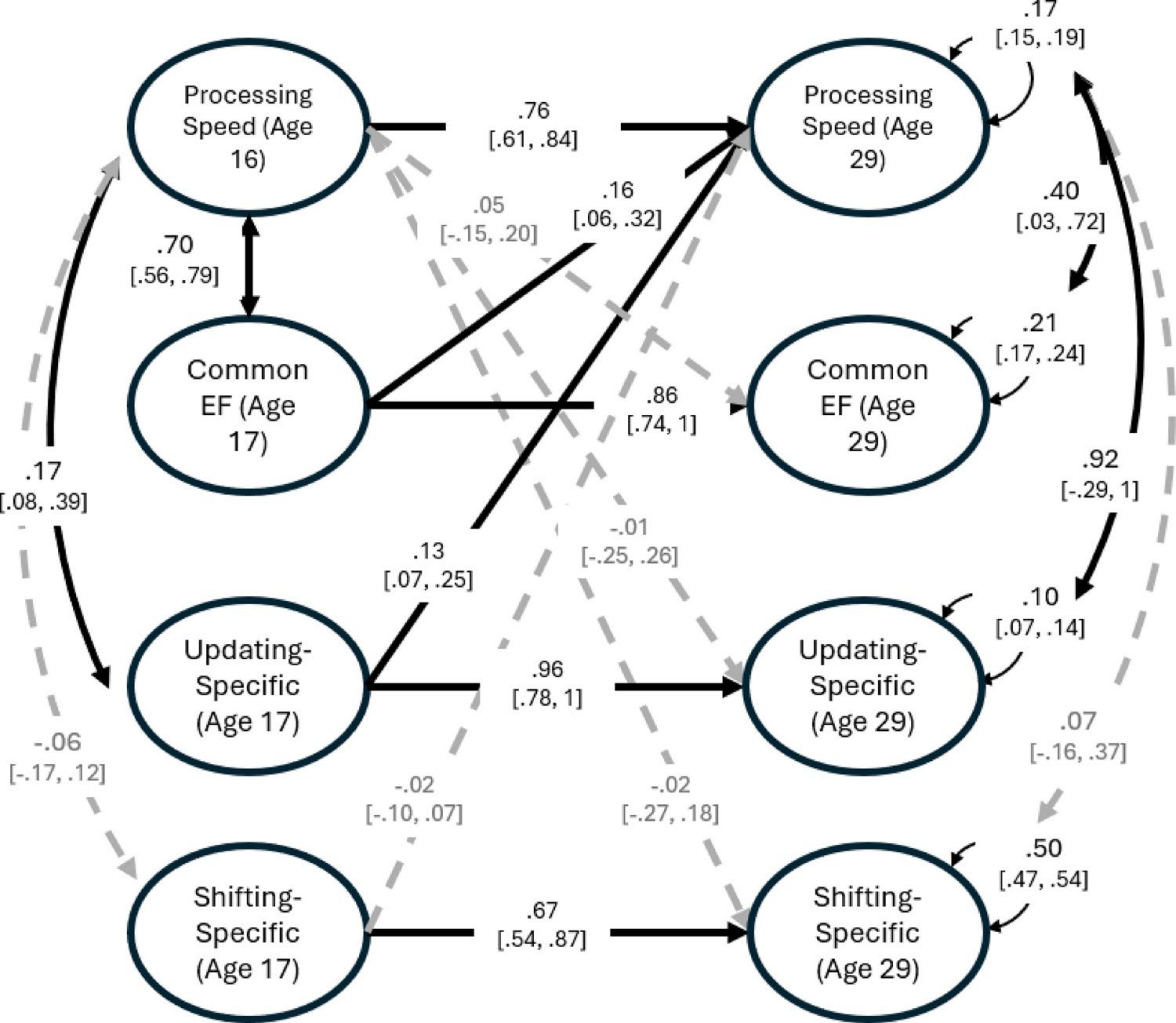
Cross-Lag model of processing speed and executive functions across late adolescence and established adulthood. Latent variables (ovals); curved arrows indicate correlations; single-headed arrows demonstrate predicted variance, in the direction of the arrowhead. Significance indicated by solid, black lines and text (*p* <.05), non-significance indicated by dashed grey lines and text (*p* >.05). Standardized associations are displayed. For simplicity, we do not display factor loadings on latent factors, or residual correlations among individual measures across time (see Fig. S2). Model fit: χ^2^ (163) = 386.926, *p* <.001. CFI = 0.957, RMSEA = 0.042

Next, Fig. 2 presents a cross-lag structural equation model examining the longitudinal relationships between PS and the three EF constructs (Common EF, Updating-Specific, and Shifting-Specific) from late adolescence (ages 16–17) to established adulthood (age 29). This model enabled us to examine whether variance in PS in late adolescence predicted new variance in EF abilities (and vice-versa), controlling for performance at baseline. Importantly, although PS in late adolescence did not predict new variance in EF factors (βs = − 0.02 to 0.05), both Common EF (β = 0.16, 95% CI [0.06, 0.32]) and Updating-specific at age 17 (β = 0.13, 95% CI [0.07, 0.25]) predicted significant new variance in PS in adulthood. Specifically, greater Common EF and Updating-specific ability in adolescence was associated with faster PS in adulthood, after controlling for PS in adolescence. Shifting-specific did not predict new variance in PS. Finally, residual variance in PS in adulthood was associated with Common EF (*r* =.40, 95% CI [0.03, 0.72]) and Updating-specific (*r* =.92, 95% CI [−0.29, 1.0]).

### Genetic Analyses

#### Stability of Genetic and Environmental Influences on Processing Speed

**Fig. 3:**
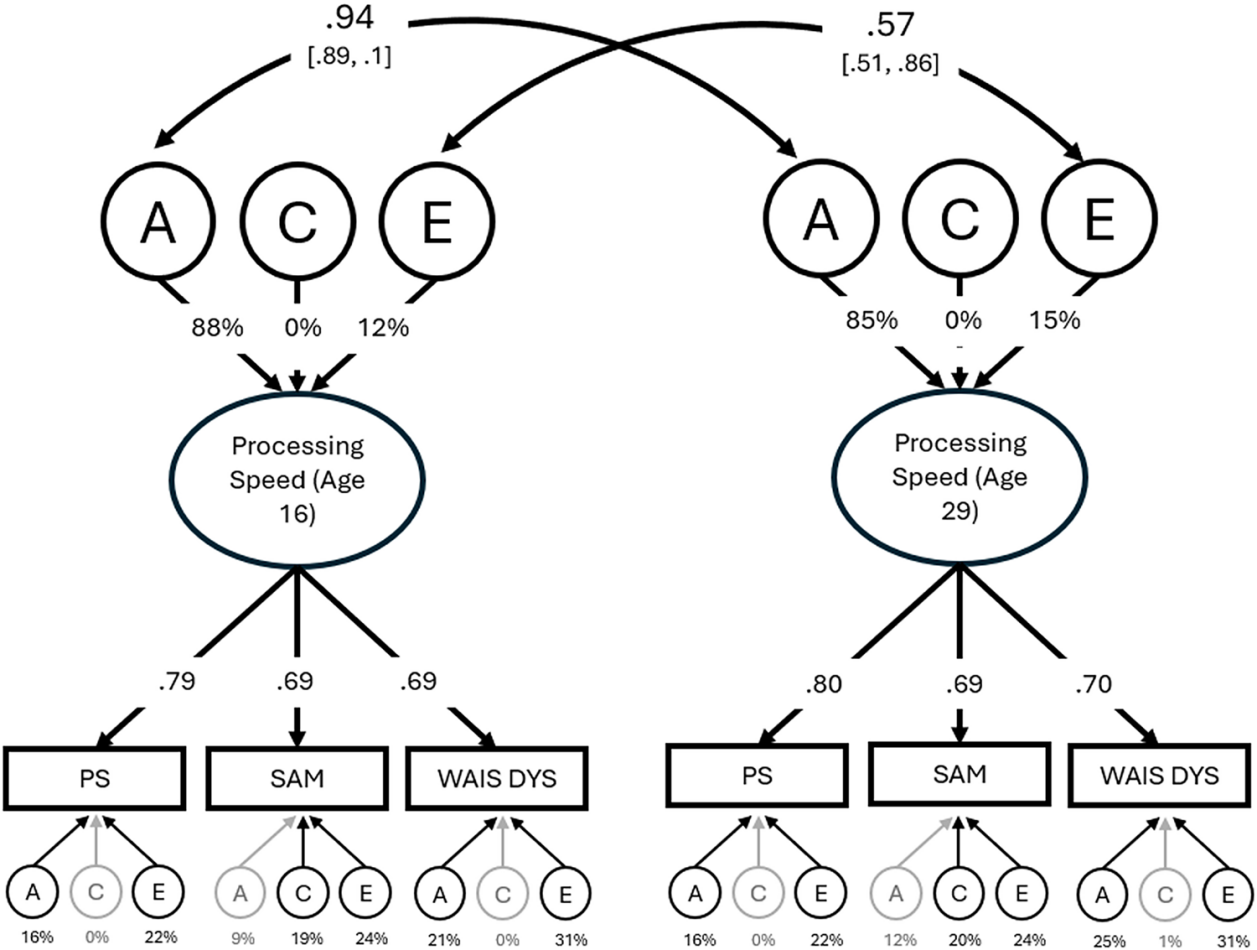
Genetic decomposition of processing speed across time. A = additive genetic influences, C = shared environmental influences, E = non-shared environmental influences. Significance indicated by solid, black lines (*p* <.05), non-significance indicated by grey lines (*p* >.05). 95% CIs displayed for genetic and non-shared environmental correlations between the latent variables (ovals). Not displayed are the residual genetic and environmental correlations among individual PS tasks. PS = Colorado Perceptual Speed, SAM = Subtract and Multiply, WAIS DYS = Wechsler Adult Intelligence Scale Digit Symbol. Model fit: χ^2^ (170) = 163.723, *p* =.621. CFI = 1.00, RMSEA = 0.00

Results from the longitudinal ACE model of PS (displayed in Fig. 3) indicated that genetic influences (A) accounted for a substantial portion of the variance in PS at both time points: a^2^ PS_age16_ = 0.88 (95% CI [0.79, 0.95]); a^2^ PS_age29_ = 0.85 (95% CI [0.74, 0.93]). There was no evidence of shared environmental contributions (C) at either timepoint, while non-shared environmental influences (E) explained the remaining 12% (95% CI [0.05, 0.21]) of the variance in PS in late adolescence and the remaining 15% (95% CI [0.07, 0.26]) of the variance in PS in established adulthood. The analysis also revealed a high degree of genetic stability in PS over time, with a genetic correlation (*r*_A_) of 0.94 (95% CI [0.87, 1.0]) between ages 16 and 29. We also observed a significant correlation between the non-shared environmental influences on PS across time: *r*_E_ = 0.57 (95% CI [0.39, 0.94]). This suggests that the same genetic factors, and similar non-shared environmental factors, largely influenced PS across both stages of the lifespan. Overall, the general PS factor demonstrated strong heritability at each age, with minimal contribution from individual-specific environmental factors.

#### Genetic and Environmental Associations Between Processing Speed and Executive Functions

**Fig. 4:**
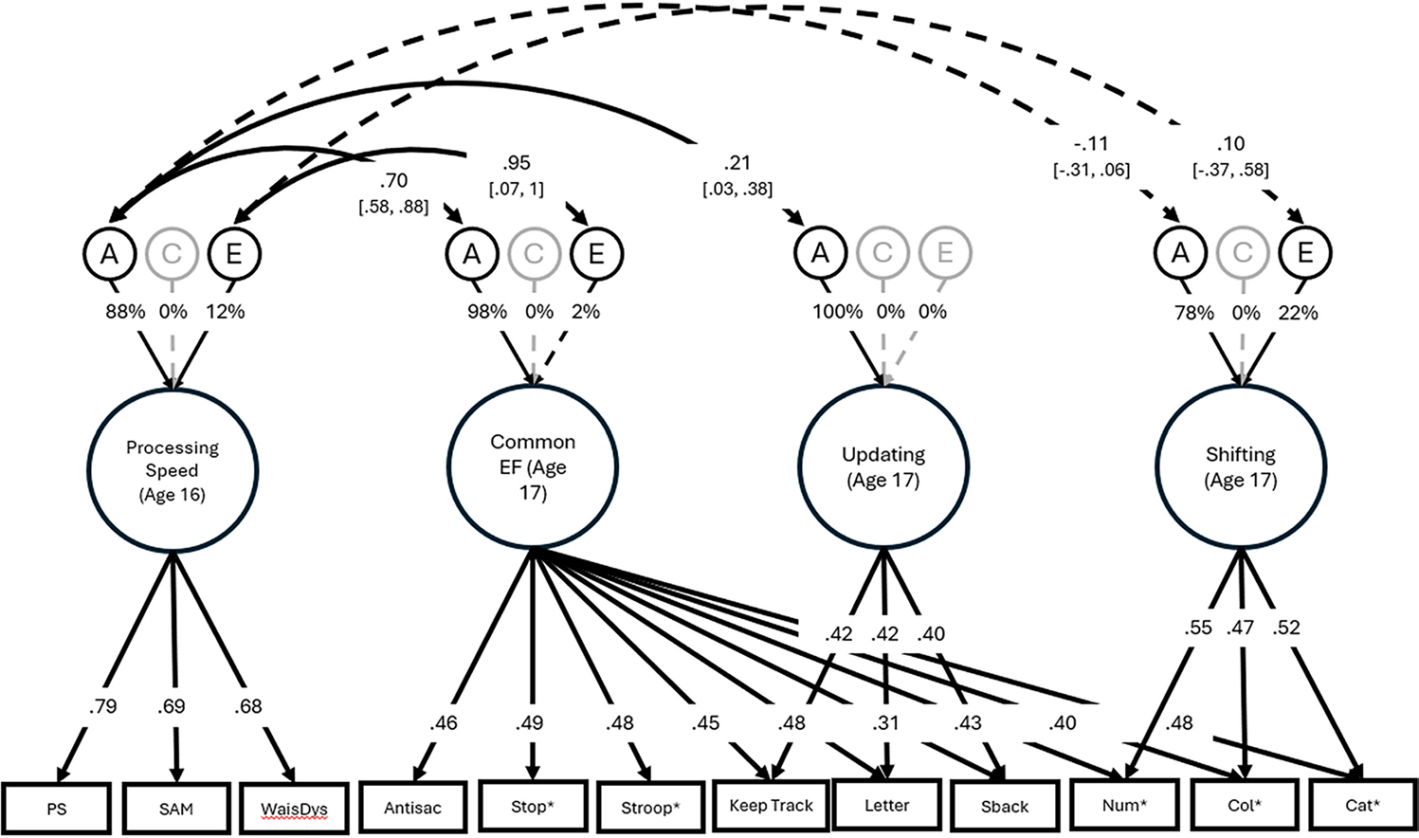
Genetic decomposition of processing speed and executive functions in late adolescence. A = additive genetic influences, C = shared environmental influences, E = non-shared environmental influences. Model fit: χ^2^ (579) = 696.446, *p* <.001. CFI = 0.957; RMSEA = 0.022. Significant genetic and environmental correlations (based on 95% CIs) are displayed with solid black arrows, and non-significant correlations with dashed lines. PS = Colorado Perceptual Speed, SAM = Subtract and Multiply, WaisDys = Wechsler Adult Intelligence Scale Digit Symbol. Antisac = Antisaccade, Stop = Stop Signal, Letter = Letter Memory, Num = Number Letter, Col = Color Shape, Cat = Category Switch. * Task reverse scored

**Fig. 5:**
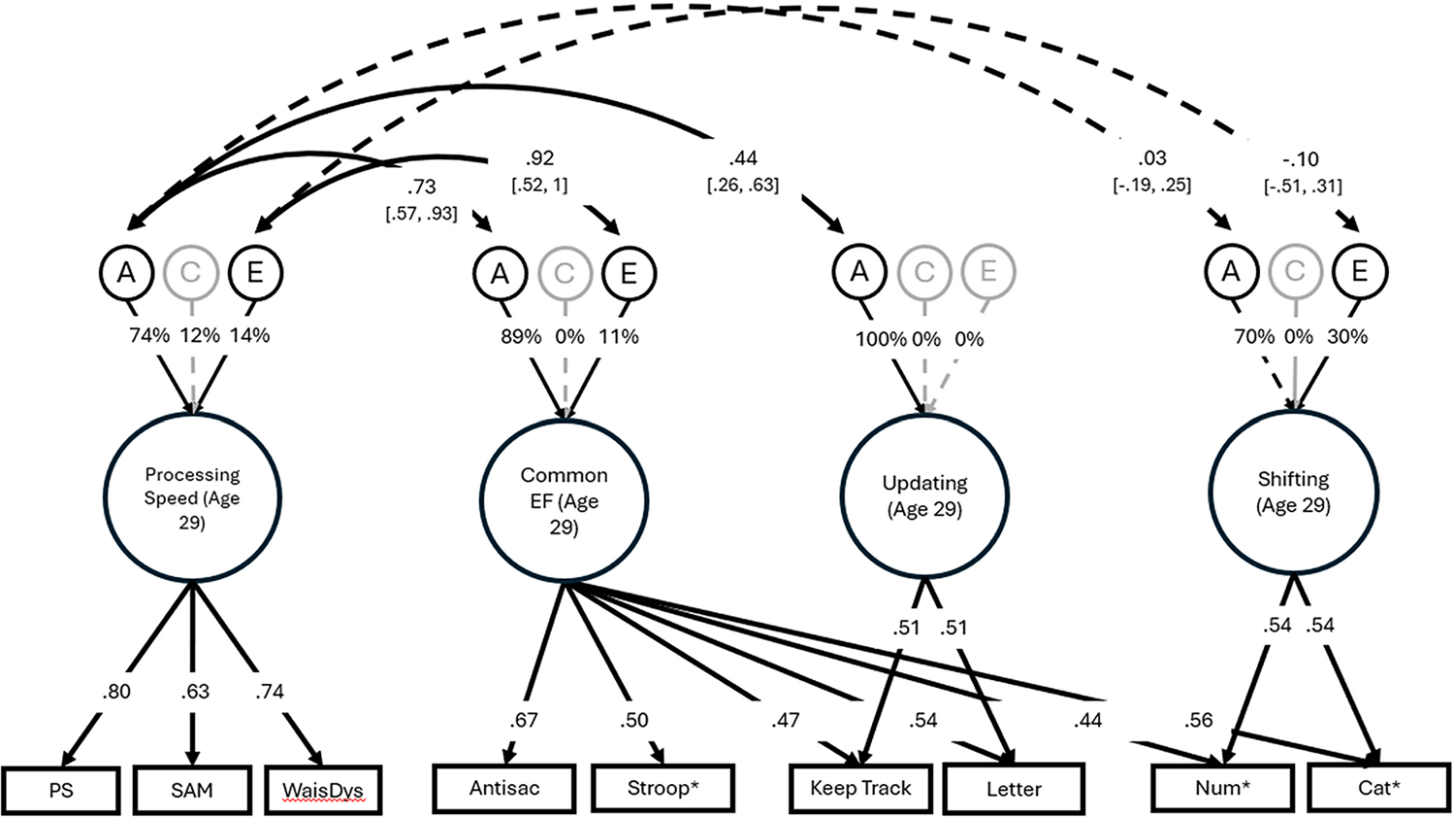
Genetic decomposition of processing speed and executive functions in established adulthood. A = additive genetic influences, C = shared environmental influences, E = non-shared environmental influences. Model fit: χ^2^ (326) = 443.080, *p* <.001. CFI = 0.948; RMSEA = 0.030. PS = Colorado Perceptual Speed, SAM = Subtract and Multiply, WaisDys = Wechsler Adult Intelligence Scale Digit Symbol. Antisac = Antisaccade, Letter = Letter Memory, Num = Number Letter, Cat = Category Switch. * Task reverse scored

ACE model estimates for the associations between PS and EFs at late adolescence (ages 16–17, Fig. 4) and established adulthood (age 29, Fig. 5) revealed distinct patterns of genetic and environmental overlap.

In late adolescence, additive genetic influences on Processing Speed (a^2^ = 0.88) were strongly correlated with those on Common EF (a^2^ = 0.98: *r*_A_ = 0.70, 95% CI [0.58, 0.88]). PS was also moderately genetically correlated with Updating-Specific abilities (*r*_A_ = 0.21, 95% CI [0.03, 0.38]), but non-significant correlations with the Shifting-Specific abilities (*r*_A_ = − 0.11, 95% CI [−0.31, 0.06]) factor. In established adulthood, the genetic correlation between PS (a^2^ = 0.74) and Common EF (a^2^ = 0.89) remained strong (*r*_A_ = 0.73, 95% CI [0.57, 0.93]). Again, there was a significant moderate genetic correlation between PS and the Updating-Specific factor (*r*_A_ = 0.44, 95% CI [0.26, 0.63]), and the genetic correlation between PS and Shifting-Specific abilities remained non-significant (*r*_A_ = − 0.10, 95% CI [−0.51, 0.31]).

Because shared environmental influences on PS and EFs were estimated to be negligible, their correlations were not estimated. Non-shared environmental influences on Processing Speed were moderately-to-strongly correlated with those on Common EF at both time points (*r*_E_ = 0.38 to 0.83), indicating moderate environmental covariation. Non-shared environmental correlations between PS and the Shifting-Specific factor remained low across time (*r*_E_ = − 0.10 to 0.10). These findings indicate that PS shares substantial genetic and non-shared environmental variance with Common EF, and that this relationship is largely stable from adolescence to adulthood. Additionally, PS was moderately genetically correlated with Updating-Specific.

## Discussion

Investigating the trajectories of PS and EFs, and their relation to one another, will be key to understanding normal, and abnormal, age-related cognitive development and decline. Using data from the LTS, we quantified the longitudinal stability of PS between late adolescence (ages 16–17) into established adulthood (age 29), including its association with multiple facets of EF as defined in the unity and diversity framework, and assessed how genetic and environmental factors contribute to these associations.

Our findings reveal high phenotypic and genetic stability of PS across these important stages of the lifespan (*r* =.90, *r*_A_ = 0.93), which is consistent with prior work demonstrating the PS is a highly stable trait with strong genetic underpinnings (Friedman and Miyake 2017a, b). The high heritability estimates (a^2^= 0.86 to 0.88) underscore the idea that individual differences in PS are largely established by late adolescence, and that there are few, if any, new genetic influences that emerge over time. Although PS is often considered a leading indicator of normal age-related cognitive decline (Salthouse 2009), we found minimal evidence for systematic decline from late adolescence to established adulthood (PS_age29_ *M* = −0.032, *p* >.05). However, without additional data it is unclear whether speed has not yet begun to decline in this sample, or if PS had not yet peaked by mean age 16 and continued to develop in the late teens and early 20 s before slightly declining in the late 20s. Non-shared environmental factors contributed modestly to the total PS variance, and demonstrated moderate temporal continuity (*r*_E_ = 0.57), suggesting that systematic interventions to maintain or enhance PS in adulthood may be most effective if they target modifiable environmental factors, even in this context of strong genetic influence.

Results from our cross-lag model indicated that new variance in PS in established adulthood was predicted by Common EF in late adolescence (β = 0.16, *p* <.05), and Updating-Specific abilities in late adolescence (β = 0.13, *p* <.05). This is indicative of directional influence whereby EF may further facilitate future PS ability, which can be linked to prior research theories in which better EF enables faster PS. These results appear to challenge the more traditional view that PS ability acts as a foundation for more complex cognitive abilities, and instead may support a bidirectional, or even EF-to-PS, model in cohorts at key developmental stages. However, given that we could not conduct a true invariance model as the EF tasks varied over time, we cannot definitively exclude the possibility that PS in adolescence predicts new variance in EFs in adulthood. As we elaborate on later, it will be important for future research to evaluate whether similar developmental associations between PS and EF are observed at earlier and later periods of the life-course.

Furthermore, we observed that Common EF also demonstrated strong phenotypic stability between late adolescence and established adulthood (*r* =.86), and was consistently correlated with PS (*r* =.70 to 0.71; see Fig. S2). Domain-specific EF components also showed strong phenotypic stability between adolescence and established adulthood (*r=*.71 to 0.96), but showed more variable correlations with PS. Specifically, the relationship between PS and Updating-Specific abilities appeared to increase with age (late adolescence: *r* =.17; established adulthood: *r* =.35), although the 95% CIs overlapped. This developmental trend suggests that working memory updating processes may become more tightly integrated with PS over time – perhaps driven by individuals acquiring more real-world cognitive demands with increased complexity. The directional increase in the phenotypic correlation between PS and the Updating-Specific factor was mirrored in their genetic correlation (late adolescence: *r*_A_ = 0.21, to established adulthood: *r*_A_ = 0.44), though again the 95% CIs overlapped, so larger samples are needed to establish whether these increases in phenotypic and genetic associations are meaningful. Conversely, Shifting-Specific abilities were consistently unrelated to PS, phenotypically and genetically, at both time points. These results reinforce the notion that not all EFs are equally associated with PS, emphasizing the need for differentiated models of cognitive function and aging (i.e., ones separating speed-linked abilities from those that are more modular/context-dependent), rather than treating cognition as a unitary construct.

Importantly, these results provide further evidence for the separability of PS and EFs. Specifically, our phenotypic findings demonstrating correlations of less than 1.0 between PS and Common EF suggest that the general processes supporting EFs are indeed distinct from PS. This is further evidenced by the fact that PS was also correlated with the orthogonal Updating-Specific factor, which was based entirely on accuracy-based tasks in the current study. These results suggest that Updating-Specific abilities, which are thought to primarily tap into working memory, also appear to include components of speed. The separability of EFs and PS was also confirmed in our ACE models in late adolescence and established adulthood, with genetic and non-shared environmental correlations (i.e., *r*A and *r*E) less than 1.0. It would be useful for future longitudinal work to examine these associations across other key developmental periods, to test whether this separability of PS and EFs is confined to this period between adolescence and established adulthood, as some suggest that PS and EFs are more unitary in childhood and later life (Cepeda et al. 2013; de Frias et al. 2009).

More broadly, understanding the distinction between PS and EF, and whether they predict one another, across other parts of the lifespan will be crucial for mapping developmental trajectories and differentiating between types of cognitive decline that may emerge later in life. Indeed, PS and EFs are some of the first cognitive abilities to decline in normal aging, and so they are prime candidates to examine as leading indicates of cognitive change in other cognitive domains. For instance, slower PS may predict broad cognitive decline (Salthouse 2000; Hartshorne and Germine 2015), but not necessarily impairments in task switching or context adaptation. Similarly, EF deficits may predict progression from mild cognitive impairment to dementia, and may do so better than other cognitive abilities like episodic memory (Junquera et al. 2020). The current study focused on cognitive changes between adolescence and young adulthood and found that EFs predicted new variance in PS but not vice-versa. However, evaluating whether similar results are observed at other stages of development and aging, including whether PS and/or EF predict new variance cognitive abilities in midlife and beyond, will be useful in expanding lifespan models of cognitive aging and for identifying individuals at greatest risk for future cognitive decline.

### Strengths and Limitations

The longitudinal design of the study allowed us to assess developmental stability and change in key cognitive domains during critical periods of the lifespan. However, there are some limitations that are worth noting. Our analyses are limited to two time points: late adolescence (ages 16–17) and established adulthood (age 29). As such, we cannot assess if, and how, the relationships between PS and EFs evolve within this time frame, or how their association changes into midlife or older age.

PS and EFs were also assessed one year apart during adolescence (PS at age 16 and EFs at age 17), and some participants completed some EF tests a few months prior to the PS tasks at age 29. While this was a small gap, it is possible that we would have observed even stronger phenotypic and genetic associations if the tests were administered on the same day, limiting our ability to strongly conclude that their associations were significantly less than 1.0. On the other hand, the slight testing delay also helps prevent overestimation of correlations that could arise from similar testing conditions confounding the results. Despite this, the associations between PS and Common EF remained highly consistent over time.

Additionally, as the number of observed measures utilized for the latent EF factors were different over time, we were unable to conduct measurement invariance testing for the EF models, limiting our ability to directly compare EF constructs over time. Moreover, although our unity and diversity model of EF incorporated tasks spanning three types of EFs that are representative of the broader inhibitory control, working memory, and cognitive flexibility domains from other frameworks (Diamond et al., 2013), it will be important to understand whether these results generalize to other types of EF tasks. Finally, our sample was predominantly non-Hispanic White, and from a limited geographic region (within the state of Colorado), which constrains the generalizability of findings to more diverse populations and cultural contexts.

## Conclusion

This study provides clear evidence that PS is a highly stable and heritable trait from adolescence into established adulthood, with robust phenotypic and genetic correlations between PS and Common EF. Nevertheless, our findings support the view that PS and EFs are related but distinct constructs, in which PS reflects lower-level cognitive operations, while EFs encompass higher-order processes like goal maintenance and cognitive control. While PS and Common EF are strongly correlated, domain-specific EF components – particularly Shifting-Specific abilities – show minimal associations with PS, indicating that EFs are not reducible to PS alone. Notably, Updating-Specific EF shows moderate associations with PS in adulthood, suggesting some domain-specific contributions to changes in PS over time. The longitudinal associations suggest that EFs – specifically Common EF and Updating-Specific abilities – can influence future changes in PS. These results deepen our understanding of how core cognitive abilities develop and interact across the lifespan, laying the groundwork for longitudinal studies that evaluate how cognitive abilities influence one another across the lifespan. Future research extending this work into midlife and older age are needed to test whether strengthening EF earlier in life slows subsequent decline in PS or buffers age-related impairments.

## Supplementary Information

Below is the link to the electronic supplementary material.

Supplementary Material 1

## Data Availability

No datasets were generated or analysed during the current study.

## Code Availability

We report how we determined our sample size, all manipulations, and all measures in the study, and the study follows JARS (Appelbaum et al. 2018). These data are part of a larger longitudinal twin study, which includes many other measures. Specifically, all EF tasks from prior confirmatory factor analyses of the age 17 and 29 assessments were included in this paper and all measures of PS that were available at both waves were included in this analysis. Analysis code and research materials are available at https://osf.io/h7gca/?view_only=2209108368f0478f96a6b01ab7cd6233. Data were analyzed using the ‘lavaan’ and ‘OpenMx’ packages in R, version 4.5.0 (R Core Team, 2025; Rosseel 2012; Boker et al. 2011). This study’s design and its analysis were not pre-registered.
