# Supplementary material for: Genetic and Environmental Associations Between Processing Speed and Executive Functions Across Adolescence and Established Adulthood
Source: Behav Genet. 2026 Jun 1;56(4):169–83. doi: 10.1007/s10519-026-10271-3 (PMC13333102; doi:10.1007/s10519-026-10271-3)
Supplement: Supplementary file 1 — Supplementary Material 1 [file 10519_2026_10271_MOESM1_ESM.docx]

Genetic and Environmental Associations Between Processing Speed and Executive Functions across Adolescence and Established Adulthood. Karhadkar *et al*., XXXX.

Supplemental Material

| Model | χ^2^ | df | *p* | RMSEA | CFI | Δχ^2^ |
| --- | --- | --- | --- | --- | --- | --- |
| 1. Configural Invariance | 15.129 | 5 | .010 | 0.050 (90% CI [0.023, 0.080]) | 0.996 | - |
| 2. Metric Invariance | 19.475 | 7 | .007 | 0.047 (90% CI [0.020, 0.075]) | 0.995 | 5.904 (df = 2, *p* = .052) |
| 3. Scalar Invariance | 20.715 | 9 | .014 | 0.040 (90% CI [0.018, 0.063]) | 0.995 | 5.586 (df = 4, *p* = .232) |
| **4. Residual Invariance** | **28.027** | **12** | **.005** | **0.041 (90% CI [0.022, 0.069])** | **0.993** | **12.898 (df = 7, *p* = .075)** |

**Table S1** *Invariance testing for phenotypic ‘Processing Speed’ model ‘Across Ages’ (ages 16 and 29).* (1) Configural Invariance = all factor loadings, intercepts and variances are freely estimated. (2) Metric Invariance = factor loadings are fixed, all other estimates are freely calculated. (3) Scalar Invariance = factor loadings and intercepts are fixed, variances are freely estimated. (4) Residual Invariance = all factor loadings, intercepts, and residual variances are fixed. Most constrained model selected for analysis


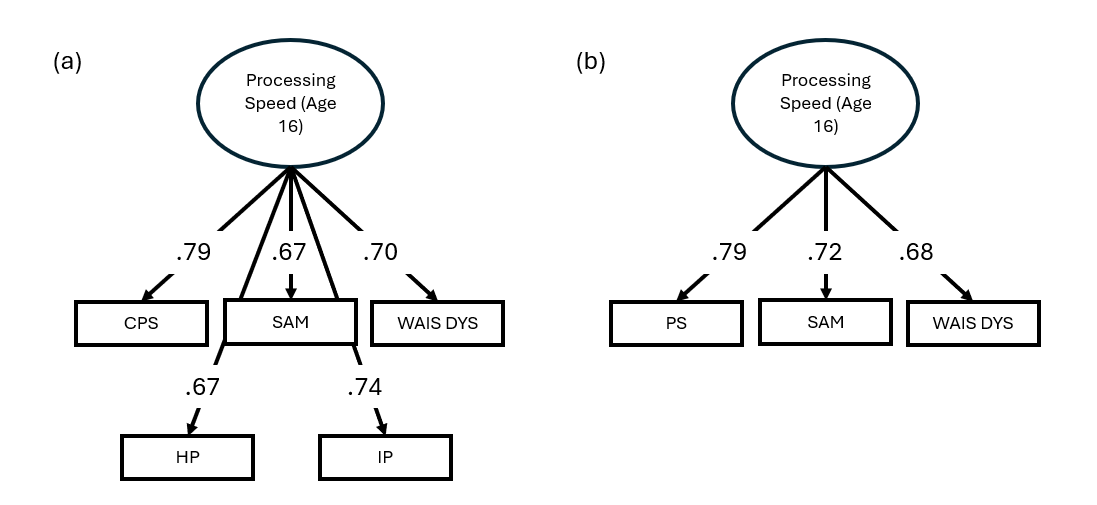
**Fig. S1** *Preliminary analyses of observed measures of Processing Speed*. (a) CPS = Colorado Perceptual Speed, HP = Hidden Patterns, SAM = Subtract and Multiply, IP = Identical Pictures, WAIS DYS = Wechsler Adult Intelligence Scale Digit Symbol. Model fit statistics: χ^2^ (5) = 74.405. CFI = 0.933, RMSEA = 0.132 [90% CI (0.109, 0.156)]. (b) Model fit statistics: χ^2^ (0) = 0.000. CFI = 1.000, RMSEA = 0.000

| Measure | 1 | 2 | 3 | 4 | 5 | 6 | 7 | 8 | 9 | 10 | 11 | 12 | 13 | 14 | 15 | 16 | 17 | 18 | 19 | 20 | 21 |
| --- | --- | --- | --- | --- | --- | --- | --- | --- | --- | --- | --- | --- | --- | --- | --- | --- | --- | --- | --- | --- | --- |
| *LTS* |  |  |  |  |  |  |  |  |  |  |  |  |  |  |  |  |  |  |  |  |  |
| 1. CPS | 1 |  |  |  |  |  |  |  |  |  |  |  |  |  |  |  |  |  |  |  |  |
| 2. SAM | .577 | 1 |  |  |  |  |  |  |  |  |  |  |  |  |  |  |  |  |  |  |  |
| 3. WAIS Digit Symbol | .562 | .504 | 1 |  |  |  |  |  |  |  |  |  |  |  |  |  |  |  |  |  |  |
| 4. Antisaccade | .173 | .065 | .199 | 1 |  |  |  |  |  |  |  |  |  |  |  |  |  |  |  |  |  |
| 5. Stop Signal | .204 | .219 | .288 | .254 | 1 |  |  |  |  |  |  |  |  |  |  |  |  |  |  |  |  |
| 6. Stroop | .308 | .251 | .251 | .159 | .134 | 1 |  |  |  |  |  |  |  |  |  |  |  |  |  |  |  |
| 7. Keep Track | .360 | .262 | .194 | .174 | .208 | .201 | 1 |  |  |  |  |  |  |  |  |  |  |  |  |  |  |
| 8. Letter Memory | .367 | .320 | .220 | .243 | .139 | .243 | .457 | 1 |  |  |  |  |  |  |  |  |  |  |  |  |  |
| 9. Spatial 2-back | .215 | .213 | .211 | .212 | .221 | .121 | .257 | .259 | 1 |  |  |  |  |  |  |  |  |  |  |  |  |
| 10. Number Letter | .164 | .139 | .260 | .137 | .236 | .224 | .130 | .186 | .148 | 1 |  |  |  |  |  |  |  |  |  |  |  |
| 11. Color Shape | .183 | .150 | .238 | .162 | .204 | .271 | .140 | .148 | .124 | .413 | 1 |  |  |  |  |  |  |  |  |  |  |
| 12. Category Switch | .175 | .146 | .222 | .210 | .265 | .247 | .170 | .156 | .196 | .475 | .425 | 1 |  |  |  |  |  |  |  |  |  |
| *CATSLife* |  |  |  |  |  |  |  |  |  |  |  |  |  |  |  |  |  |  |  |  |  |
| 13. CPS | .752 | .474 | .488 | .199 | .186 | .319 | .370 | .381 | .193 | .166 | .166 | .192 | 1 |  |  |  |  |  |  |  |  |
| 14. SAM | .534 | .790 | .425 | .135 | .211 | .199 | .336 | .320 | .195 | .147 | .164 | .167 | .521 | 1 |  |  |  |  |  |  |  |
| 15. WAIS Digit Symbol | .563 | .462 | .722 | .243 | .315 | .299 | .279 | .258 | .214 | .284 | .246 | .245 | .588 | .441 | 1 |  |  |  |  |  |  |
| 16. Antisaccade | .248 | .148 | .264 | .473 | .250 | .259 | .228 | .243 | .142 | .294 | .226 | .255 | .304 | .192 | .346 | 1 |  |  |  |  |  |
| 17. Stroop | .310 | .270 | .291 | .153 | .188 | .434 | .201 | .203 | .178 | .165 | .128 | .257 | .334 | .204 | .341 | .238 | 1 |  |  |  |  |
| 18. Keep Track | .362 | .284 | .245 | .188 | .222 | .158 | .494 | .369 | .217 | .162 | .152 | .162 | .439 | .350 | .389 | .328 | .226 | 1 |  |  |  |
| 19. Letter Memory | .409 | .371 | .255 | .241 | .187 | .282 | .483 | .507 | .253 | .178 | .166 | .192 | .477 | .423 | .341 | .383 | .295 | .499 | 1 |  |  |
| 20. Number Letter | .150 | .104 | .259 | .187 | .157 | .200 | .133 | .121 | .126 | .533 | .310 | .426 | .191 | .069 | .316 | .320 | .273 | .154 | .137 | 1 |  |
| 21. Category Switch | .248 | .192 | .312 | .214 | .267 | .305 | .230 | .183 | .156 | .416 | .317 | .538 | .279 | .201 | .345 | .386 | .316 | .240 | .229 | .536 | 1 |

**Table S2** *Pearson correlations between PS and EF tasks at ages 16-17 and 29, prior to scaling for analysis.* LTS = Longitudinal Twin Study, CPS = Colorado Perceptual Speed, SAM = Subtract and Multiply, WAIS = Wechsler Adult intelligence Scale, CATSLife = Colorado Adoption/Twin Study of Lifespan Behavioral Development and Cognitive Aging. Significance for all correlations are *p* < .001, with the exception of CATSLife Number Letter ~ SAM, which was marginally significant (*p* = .082). All CATSLife EF measures are harmonized

**
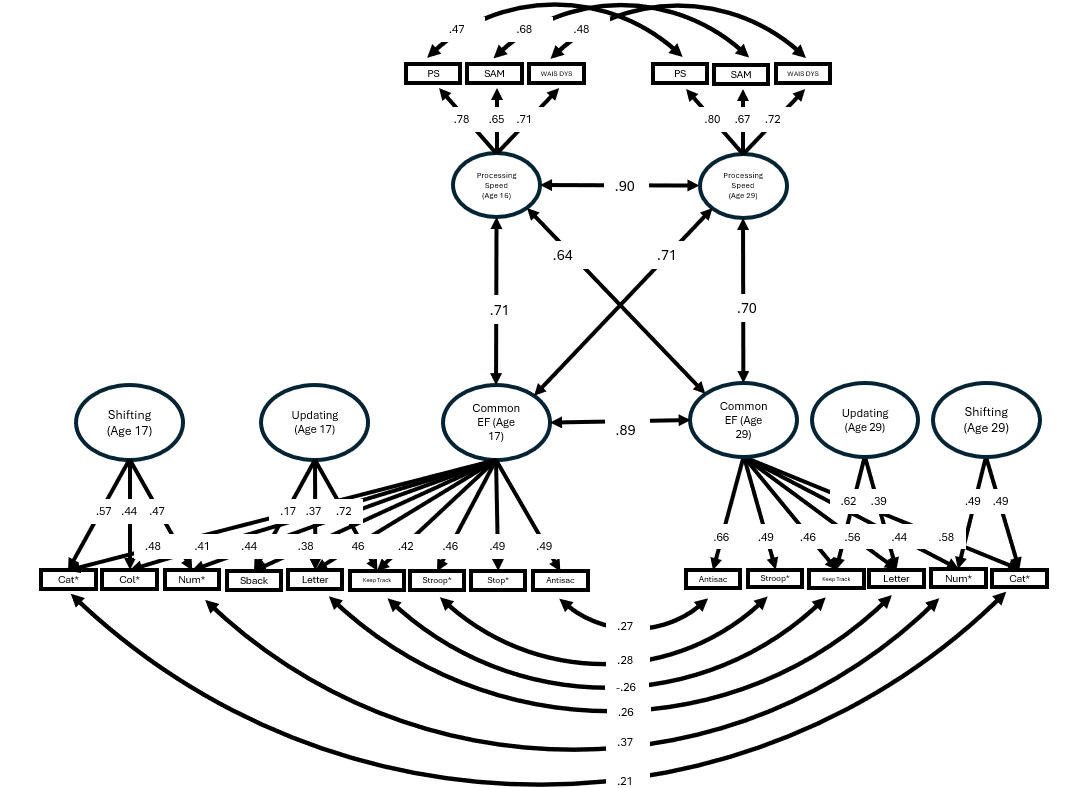
**

**Fig. S2** *Full* *SEM Path Diagram for Correlation Matrix between Processing Speed and Executive Functions across Late Adolescence and Established Adulthood.* PS = Colorado Perceptual Speed, SAM = Subtract and Multiply, WAIS DYS = Wechsler Adult Intelligence Scale Digit Symbol. Antisac = Antisaccade, Stop = Stop Signal, Letter = Letter Memory, Num = Number Letter, Col = Color Shape, Cat = Category Switch. Model fit statistics : χ^2^ = 355.185 (df = 163, *p* < .001); CFI = 0.963; RMSEA = 0.038 (90% CI [0.033, 0.043]). * Task was reverse score in keeping with theoretical interpretation
